# Molecular Detection of *Coxiella*-like Endosymbionts in Ticks in Hebei, China

**DOI:** 10.3390/pathogens15060647

**Published:** 2026-06-18

**Authors:** Ze-Yun Xu, Guo-Qing Chen, Jing Xue, Yu-Xin Chi, Rui Jian, Wen-Ping Guo

**Affiliations:** 1 College of Basic Medicine, Chengde Medical University, Chengde 067000, China; a451583920@gmail.com (Z.-Y.X.);; 2 Key Laboratory of Public Health Molecular Biology, Yancheng Center for Disease Control and Prevention, Yancheng 224001, China

**Keywords:** tick, *Coxiella*-like endosymbiont, *IS1111*, 16S rRNA, *groEL*, *rpoB*

## Abstract

Ticks are widely distributed in China and can carry and transmit a variety of pathogens that potential to cause serious impacts on public health and the economy. Little is known about the broader spectrum of *Coxiella*-like endosymbiont (CLE) in ticks under natural conditions in China. The aim of this study was to detect, analyze, and characterize phylogenetically CLE found in ticks in Hebei Province, China. A total of 947 ticks collected from Hebei Province were identified as *Haemaphysalis longicornis* based on morphological characteristics and *cytochrome c oxidase* gene PCR analysis of extracted DNA. Subsequently, DNA was analyzed via PCR for the *IS1111* gene (frequently associated with *Coxiella burnetii*), and the amplified DNA was then sequenced and analyzed phylogenetically using a set of primers targeting the 16S rRNA, *groEL*, and *rpoB* genes. A total of 8.24% (78/947) of ticks from the Chengde, Baoding, and Cangzhou regions were positive in the *IS1111* PCR. Phylogenetic analysis using the 16S rRNA, *groEL*, and *rpoB* genes revealed the presence of CLE in *Ha. longicornis* ticks from these regions and the formation of two distinct clades, suggesting horizontal gene transfer events. Our results strengthen the growing evidence that CLE, not *Coxiella burnetii*, is ubiquitously associated with ticks across diverse geographic locations—a distinction critical for accurately interpreting tick microbiome surveys and avoiding false assumptions of zoonotic risk.

## 1. Introduction

*Coxiella* is a monotypic genus of obligate intracellular Gammaproteobacteria belonging to the family *Coxiellaceae*, order *Legionellales* [1]. Members of the *Coxiella* genus are globally distributed bacteria with a diversity of hosts, including mammals, birds, reptiles, amphibians, and arthropods [2]. Within this genus, *Coxiella burnetii*—a Gram-negative, intracellular, parasitic bacterium [3]—is the established causative agent of the global zoonotic disease Q fever [1]. *Coxiella burnetii*, commonly found in small domestic ruminants, such as sheep, goats, and cattle, is considered to be a major source of human infection [4,5].

However, there are also other bacteria called *Coxiella*-like or *Coxiella* sp., which are considered to be endosymbionts of ticks [6]. A recent hypothesis suggests that both *C. burnetii* and the aforementioned endosymbionts evolved from a single, virulent common ancestor, with some lineages subsequently losing the ability to infect vertebrate hosts [7]. Phylogenetic analyses based on multi-locus sequencing and whole genomes indicate that all known strains of *C. burnetii* form a distinct lineage within the broader diversity of *Coxiella*-like bacteria and share a common ancestor with tick-associated *Coxiella*-like endosymbionts (CLEs) [8]. Furthermore, an increasing number of studies, primarily using 16S rRNA gene sequencing, have identified diverse novel *Coxiella*-like organisms in ticks. Many of these act as commensal or mutualistic symbionts for their tick hosts [2]. Based on 16S rRNA phylogenies, CLEs form a monophyletic clade with the pathogen *C. burnetii*, indicating their close evolutionary relationship. Compared to *C. burnetii*, however, these endosymbionts typically possess a reduced genome [9].

Ticks are obligate ectoparasites that must feed on host blood during some or all stages of their life cycle. Pathogens transmitted by ticks constitute an emerging global public health concern, posing a threat to both humans and animals [10]. Several studies suggest that ticks play a crucial role in the maintenance of the natural cycle and in the transmission of *C. burnetii* [8,11] and CLEs, as well as a variety of other pathogens [9]. To date, a variety of hard and soft tick species have been documented as hosts for *C. burnetii*, including *Haemaphysalis*, *Amblyomma*, *Rhipicephalus*, *Hyalomma*, and *Dermacentor* [12,13,14]. CLEs—the most common symbionts of ticks—have been reported in diverse hard and soft tick genera (e.g., *Haemaphysalis*, *Amblyomma*, *Rhipicephalus*, *Ixodes*, and *Ornithodoros* [15]); moreover, strains closely related to *C. burnetii* have been isolated from ticks, with growing evidence suggesting their association with human bacteremia [2]. *Coxiella burnetii* can cause human bacteremia, with potential clinical consequences including chronic Q fever, endocarditis, and hepatitis [2]; meanwhile, the potential role of CLE in bacteremia remains a topic of growing concern.

Human cases of Q fever have been documented in Hebei Province of China, whereas no published data concerning the detection of *Coxiella burnetii* in local tick populations is available to date. Therefore, understanding the local tick-borne presence of *C. burnetii* and CLE is critical to prevent Q fever. In this study, ticks from Hebei Province were screened for CLEs. This study aimed to update the information on the presence of CLEs and to identify and genetically characterize *Coxiella* in ticks collected from several prefecture-level city areas in Hebei Province, China.

## 2. Methods

### 2.1. Collection and Identification of Ticks and DNA Extraction

**Figure 1:**
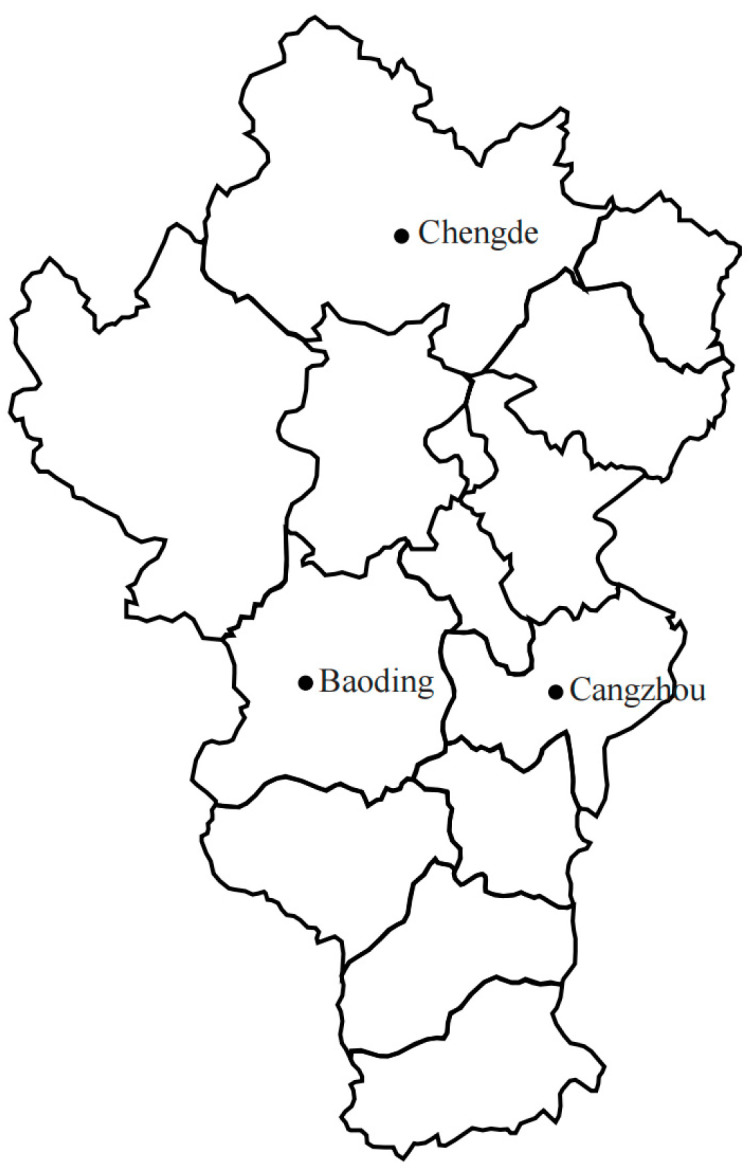
Map with the location of the collection site of ticks (●) in Chengde, Baoding, and Cangzhou City of Hebei Province, China.

A total of 947 ticks were collected in the natural environments of Chengde City (n = 623), Baoding City (n = 204), and Cangzhou City (n = 120), Hebei Province (Figure 1), by the artificial cloth flagging method [10], identified morphologically according to their appearance, and stored at −80 °C. Initially, morphological characteristics were used to identify ticks at the species level. In addition, the tick species was further identified by polymerase chain reaction (PCR) analysis of the cytochrome c oxidase I (*COI*) gene sequence [16].

All collected tick specimens were washed three times with 75% alcohol and then twice with phosphate-buffered saline (PBS) solution. Total DNA was extracted from each tick with the Tissue DNA Extraction Kit (Omega, Norcross, GA, USA) following the manufacturer’s protocol. The extracted DNA was eluted in 80 μL double-distilled water (ddH_2_O) and stored at −80 °C. Table 1 lists all primers used in this study.

### 2.2. Molecular Identification of Coxiella by the IS1111 Gene

The tick DNA samples were examined for *C. burnetii* by nested PCR (nPCR) and by sequencing the *IS1111* gene with *C. burnetii*-specific primers. The first round of nPCR utilized primer pair QBT1/QBT2 [17], while the second round employed QBTN3/QBTN4 [18].

### 2.3. Molecular Characterization of Coxiella of 16S rRNA, groEL, and rpoB Gene

To obtain additional DNA sequences for phylogenetic analyses, additional nPCR assays for the 16S rRNA, *groEL*, and *rpoB* genes were performed on all of the *IS1111*-positive tick DNA.

To better understand the genetic characteristics, a partial 16S rRNA gene was amplified by nPCR from *IS1111*-positive samples. The first round of nPCR used primer pair Cox16S-F1/16S-R, and the second round used primer pair 16S-F/16S-R [19].

For genetic characterization, a partial *groEL* gene was amplified by nPCR from *IS1111*-positive samples using primer pairs CoxGrF1/CoxGrR2 (first round) and CoxGrF2/CoxGrR1 (second round) [20].

A partial *rpoB* gene was amplified by nPCR from *IS1111*-positive samples for genetic characterization using primer pairs CoxrpoBF2/CoxrpoBR1 (first round) and CoxrpoBF3/CoxrpoBR3 (second round) [20].

To avoid contamination, DNA extraction, PCR mix preparation, template addition, PCR amplification, and electrophoresis were carried out in separate rooms under fume hoods, and all operations used dedicated pipettes and filter tips. In addition, ddH_2_O replaced DNA in the negative control assays.

### 2.4. Sequencing and Nucleotide Sequence Analysis

PCR products were analyzed by electrophoresis on 1% agarose gels for *IS1111*, 16S rRNA, *groEL*, and *rpoB* genes. All PCR products of the expected size were selected, purified, and cloned into pMD19-T vectors (Takara, Dalian, China). All DNA sequences obtained in this study were determined by Sanger sequencing using the ABI-PRISM Dye Terminator Sequencing Kit and the ABI 3730 Genetic Analyzer, with the universal primer pair M13-47 (forward) and RV-M (reverse) of T-vector (Sangon, Beijing, China).

BioEdit version 7.1.11 was used to edit all newly generated sequences in this study [21]. The obtained *IS1111* gene, 16S rRNA, *groEL* ,and *rpoB* gene sequences were analyzed using BLASTn on the NCBI website. Similarity analyses were performed with CLUSTAL W. The *IS1111* gene, 16S rRNA, *groEL*, and *rpoB* gene sequences of the most closely related species were retrieved from GenBank and aligned.

Nucleotide sequence similarities were calculated with the MegAlign program within the Lasergene software suite [22]. To further explore the phylogenetic relationship between the CLEs obtained in this study and other known strains, a maximum likelihood (ML) tree was constructed based on the 16S rRNA, *groEL*, and *rpoB* gene sequences using MEGA version 6.0.6 [23]. Using the same software, the general time-reversible (GTR) model was selected as the best-fit nucleotide substitution model, incorporating a gamma distribution (G) and a proportion of invariable sites (I), i.e., GTR + G + I [23]. Bootstrap support was assessed with 1000 replicates, and the resulting phylogenetic trees were midpoint-rooted for better interpretation.

## 3. Results

We have compiled the PCR-positive results for 16S rRNA, *groEL*, and *rpoB* genes of CLE (Table 2).

### 3.1. Identification of Ticks

**Figure 2:**
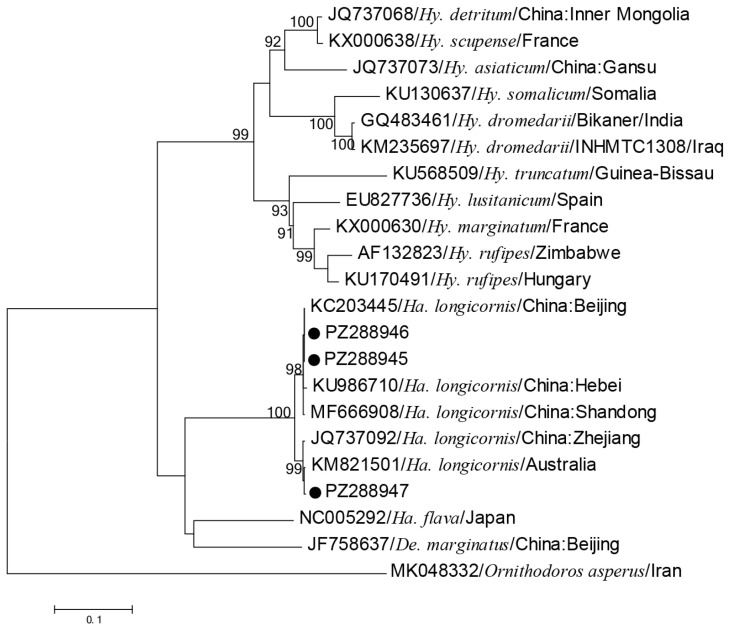
Molecular identification of ticks based on the phylogenetic analysis of the *COI* gene. The maximum likelihood (ML) tree was reconstructed using MEGA 6.0.6 under the GTR + G + I model with 1000 replicates. The numbers at each node indicate bootstrap values, and only bootstrap values > 70% are shown at appropriate nodes. Taxa marked by a circle depict the representative sequence obtained in this study. Sequence from *Ornithodoras asperus* (MK048332) was included as the outgroup.

The *COI* phylogenetic tree illustrates the genetic relationships among *COI* genes in different tick species. The COI genes of tick species from the three locations studied in this research all clustered together with those of the known tick species *Ha. longicornis* (KC203445, KU986710, MF666908, JQ737092, KM821501). The sequence identity between the *COI* genes from these locations and those of *Ha. longicornis* is 97.6–100%. All ticks collected in this study were identified as *Ha. longicornis* (Figure 2) based on the cytochrome c oxidase I (COI) gene sequences obtained by polymerase chain reaction (PCR) [16].

### 3.2. Molecular Characterization of the IS1111 Gene of Coxiella

Out of 947 tick specimens, 78 were found to carry the *IS1111* gene with a positivity rate of 8.24% (78/947), and it was identified that the tick-borne *Coxiella* showed 99.74–100% homology with the *IS1111* sequences retrieved from the Genbank database (GenBank numbers: OM654093, MT900501, PX422556). Homology results were between 99.7 and 100% in 78 positive specimen sequences, and all the *IS1111* gene sequences obtained in this study were submitted to GenBank under the accession numbers PZ276418-PZ276495.

### 3.3. Molecular Characterization of the 16S rRNA Gene of Coxiella

Furthermore, 16S rRNA was successfully amplified in 56 out of 78 *IS1111*-positive specimens, and it was determined that the tick-borne CLE showed 95.99–100% homology to the 16S rRNA sequence retrieved from the Genbank database (GenBank numbers: LC635187, MZ047981, KP994813). The homology results of the 56 positive specimen sequences ranged from 96.2 to 100. The 16S rRNA gene sequences obtained in this study were submitted to GenBank under the accession numbers PZ269377-PZ269432.

**Figure 3:**
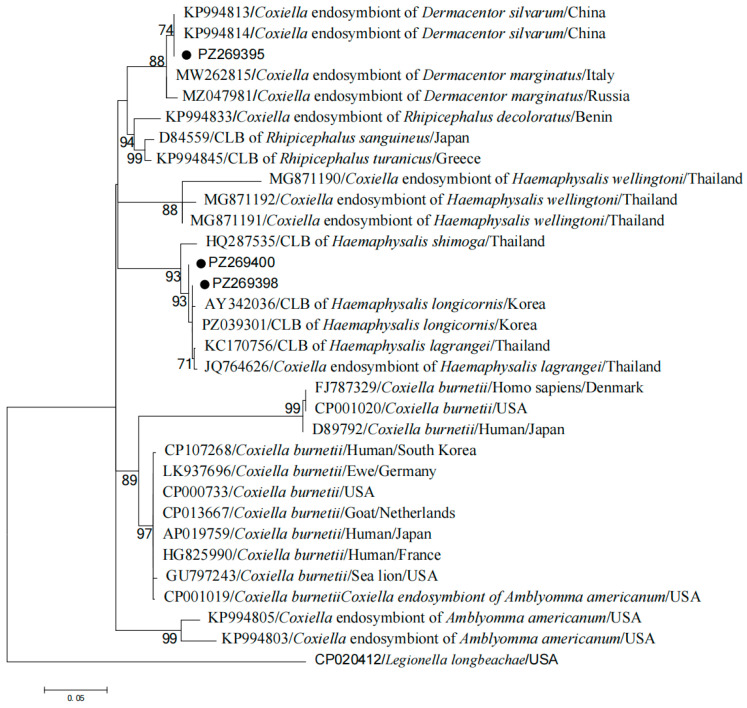
Phylogenetic tree of CLE based on partial 16s rRNA sequences. The numbers at each node indicate bootstrap values, and only bootstrap values > 70% are shown at appropriate nodes. Taxa marked by a circle depict the representative sequence obtained in this study. The sequence from *Rickettsia melolonthae* (CP020412) was included as the outgroup.

The 16S rRNA gene tree showed genetic evolutionary relationships among different strains of CLE. The 16S rRNA gene tree showed that the 16S rRNA gene of CLE in ticks obtained in this study clustered together with other known CLE strains and were separated from two other classes of CLE strains (Figure 3). All characterized sequences detected in this study belonged to two endosymbiotic groups. PZ269395 clustered with the CLE of *Der. silvarum* found in China (KP994813, KP994814) and the CLE of *Der. marginatus* found in Italy (MW262815) and Russia (MZ047981). In addition, PZ269398 and PZ269400 clustered with the CLE of *Ha. lagrangei* isolated in Thailand (KC170756) and the CLE of *Ha. longicornis* isolated in Korea (PZ039301 and AY342036) (Figure 3). Consistently, PZ269395 shared a 98.6–100% nucleotide identity with KP994813, MW262815, and MZ047981 for the partial 16S rRNA gene; PZ269398 and PZ269400 shared a 99.5–100% nucleotide identity with KC170756, PZ039301, and AY342036.

### 3.4. Molecular Characterization of the groEL Gene of Coxiella

The *groEL* gene was successfully amplified in 53 out of 78 *IS1111*-positive specimens, and it was determined that the tick-borne CLE showed 78.15–100% homology to the *groEL* gene sequence retrieved from the Genbank database (GenBank numbers: KY678195, KP985496, OR060696). The homology results of the 53 positive specimen sequences ranged from 76.9 to 100. The *groEL* gene sequences obtained in this study were submitted to GenBank under the accession numbers PZ276496-PZ276548.

**Figure 4:**
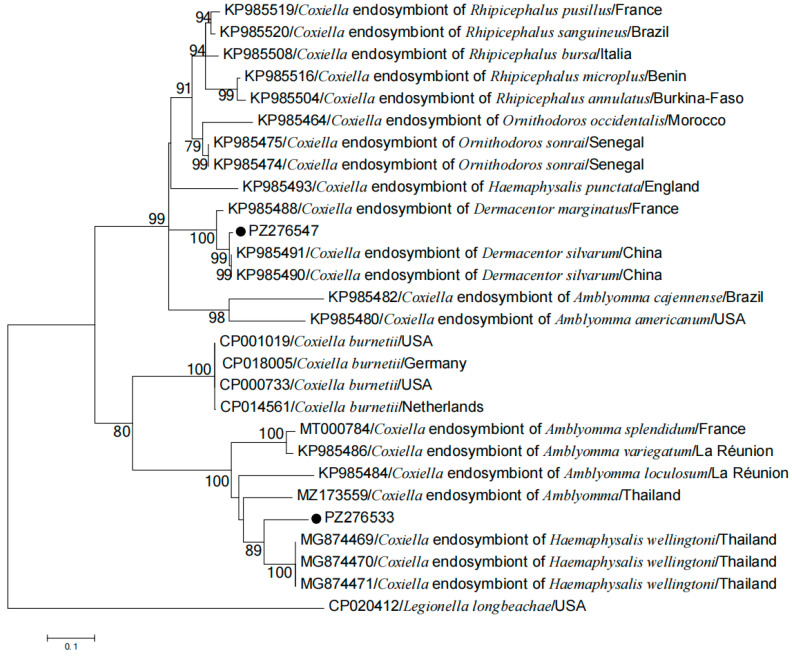
Phylogenetic tree of CLE based on the partial *groEL* sequences. The numbers at each node indicate bootstrap values, and only bootstrap values > 70% are shown at appropriate nodes. Taxa marked by a circle depict the representative sequence obtained in this study. The sequence from *Legionella longbeachae* (CP020412) was included as the outgroup.

The *groEL* gene tree showed genetic evolutionary relationships among different strains of CLE. The *groEL* gene tree showed that the *groEL* gene of CLE in ticks obtained in this study clustered together with other known CLE strains and separated from two classes of CLE strains (Figure 4). PZ276547 clustered with the CLE of *Der. Silvarum* was found in China (KP985490 and KP985491), and the genetic similarity between them is 98.9–100%; PZ276533 clustered with the CLE of *Ha. wellingtoni* (MG874471, MG874469, MG874470) found in Thailand, and they shared 89.1% nucleotide identity with each other.

### 3.5. Molecular Characterization of the rpoB Gene of Coxiella

The *rpoB* gene was successfully amplified in 58 out of 78 *IS1111*-positive specimens, and it was determined that the tick-borne CLE showed 82.54–99.79% homology to the *rpoB* gene sequence retrieved from the Genbank database (GenBank numbers: KP985328, OU015521, PQ295825). The homology results of the 58 positive specimen sequences ranged from 74.9 to 100. The *rpoB* gene sequences obtained in this study have been submitted to GenBank under the accession numbers PZ276549-PZ276606.

**Figure 5:**
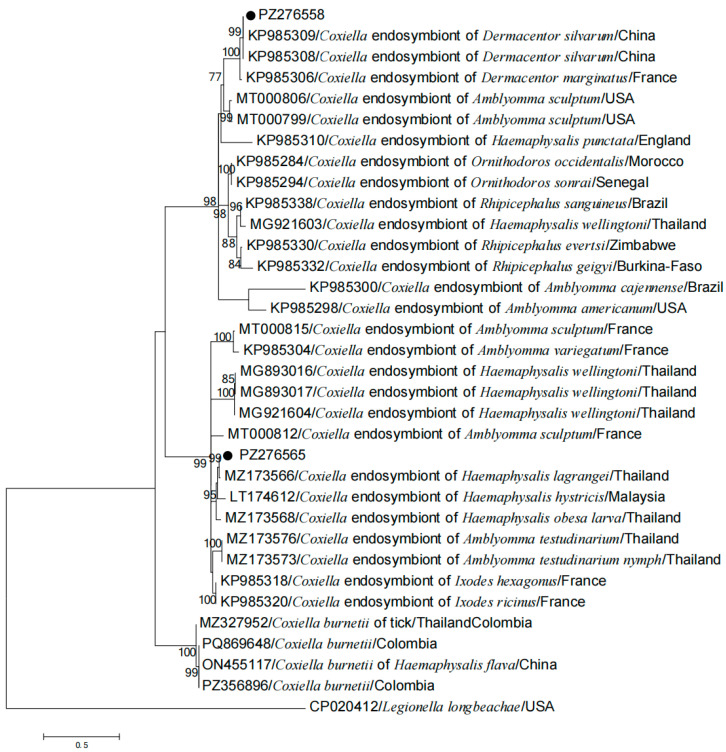
Phylogenetic tree of CLE based on partial *rpoB* sequences. The numbers at each node indicate bootstrap values, and only bootstrap values > 70% are shown at appropriate nodes. Taxa marked by a circle depict the representative sequence obtained in this study. The sequence from *Legionella longbeachae* (CP020412) was included as the outgroup.

The *rpoB* gene tree showed genetic evolutionary relationships among different strains of CLE. The *rpoB* gene tree showed that the *rpoB* gene of CLE in ticks obtained in this study clustered together with other known CLE strains and separated from two classes of CLE strains (Figure 5). PZ276558 clustered with the CLE of *Der. silvarum* (KP985308, KP985309) found in China, and the genetic similarity between them is 100%; PZ276565 clustered with the CLE of *Ha. lagrangei* (MZ173566) found in Thailand, and they shared 98.7% nucleotide identity with each other.

As shown in Figure 3, Figure 4 and Figure 5, the CLE strains identified in the present study fell into two distinct groups. In the phylogenetic trees of the 16S rRNA, *groEL*, and *rpoB* genes, all the CLEs recovered from ticks in Cangzhou clustered together with previously reported CLEs found in Der. silvarum from other regions of China, indicating the closest genetic relationship among them, although identified from different tick species. However, another group of CLE strains clustered together with different reference strains across the three gene trees. This topological discrepancy results from the fact that the reference strains in GenBank lack sequence data for all three target genes.

## 4. Discussion

The genus *Coxiella* comprises obligate intracellular bacteria that share biological characteristics with members of the order *Rickettsiales* [7]. The genus contains two recognized species (*C. burnetii* and *C. cheraxi*), one *Candidatus* species (*Candidatus Coxiella mudrowiae*), and several unrecognized *Coxiella* symbionts [24]. *Coxiella burnetii* is the only recognized pathogenic species, responsible for the global zoonotic disease Q fever [25]. People infected with *C. burnetii* typically present with flu-like symptoms (e.g., high fever, severe headache, fatigue, and night sweats) one to three weeks after exposure, along with possible diarrhea, abdominal pain, or chest pain [4]. Some *Coxiella*-like bacteria have also been identified as human pathogens; these infections are associated with a scalp eschar and cervical lymphadenopathy and can lead to various systemic complications [26,27]. Furthermore, infection with CLEs can be fatal in some animals, including birds [28]. Pathological findings in such cases include multifocal hepatic necrosis with infiltration by mixed inflammatory cells [28] as well as myocardial degeneration and necrosis [29].

Symbionts are found in a wide range of insect hosts, where they provide essential benefits such as aiding in development, feeding, and reproduction, as well as enhancing defense against natural enemies, environmental stress, and improving immunity [30]. Ticks are widely distributed throughout the world and, after mosquitoes, are one of the most important vectors of human disease and the main carriers of pathogens in wildlife and domestic animals [31]. *Coxiella burnetii* can be carried by various hosts, including wild and domesticated mammals, birds, and ticks. Ticks, in particular, play an essential role in transmitting the pathogen between animals and are important vectors of Q fever [32,33]. The presence of CLE in arthropods, especially ticks, has been reported in related studies, and CLE may be specific endosymbionts of several tick genera, including *Ixodes*, *Amblyomma*, *Ornithodoros*, and *Rhipicephalus* [34]. In addition, in several regions of Thailand, CLEs have been reported from various tick species [9,35,36]. CLEs were found in at least 10 tick species collected from both vegetation and animals, including *Ha. lagrangei*, *Ha. shimoga*, *Der. atrosignatus*, *A. testudinarium*, *Ha. hystricis*, *Ha. bispinosa*, *Ha. obesa*, *Der. auratus*, *Ha. wellingtoni* and *Rh. microplus* [35,36,37,38,39]. Moreover, on the African continent, CLEs have been reported in a number of tick species, which include *Rh. maculatus*, *A. cohaerens*, *A. gemma*, *Hyalomma truncatum*, *A. personatum*, *A. tholloni*, *Rh. pravus*, *A. variegatum*, *Ha. leachi*, *Haemaphysalis* sp., *Rh. carnivoralis*, *Rh. appendiculatus*, *Rh. evertsi*, *Rh. praetextatus*, *Rh. compositus*, *A. lepidum*, *Rh. sanguineus*, and *Rhipicephalus* sp. [40,41]. However, little is known about the broader array of CLE in ticks under natural conditions in China. In China, CLE has been identified as *Ha. flava*. (2.37%, 5/211); *Ha. longicornis* (20%,3/15), collected from Shanghai [42]; *Ha. flava* (61.1%, 107/175) from Jiangsu [43]; *Der. nuttalli* (53.69%, 109/203) from Inner Mongolia [44]; and *Rh. microplus* (27%, 54/200) from Guizhou [45].

At the time of the original description, *IS1111* had only been found in *C. burnetii*. However, in this study, we have found that when screened with primers initially thought to be *C. burnetii*-specific, some *Coxiella*-like bacteria give positive PCR results. Not only the present work but also the earlier study by Vilcins et al. show that *IS1111* copies exist in other bacteria, specifically the CLEs of ticks [46]. In addition, our results of the *IS1111* gene are similar to the findings described by Duron et al., who previously reported that the *IS1111* gene shows a high degree of genetic diversity in *C. burnetii* and CLE [20]. The *IS1111* transposable element, which is routinely targeted in epidemiologic studies of *C. burnetii* prevalence in ticks, was the most frequently detected marker in ticks infected with CLEs [47], as demonstrated in this study. *IS1111* is widely distributed within the genus *Coxiella* and is not unique to *C. burnetii* [48]. Variant copies of this element have been identified in CLEs inhabiting distantly related tick species, including both soft ticks (e.g., *Ornithodoros*, *Argas*) and hard ticks (e.g., *Rhipicephalus*, *Haemaphysalis*) [48]. Thus, our findings demonstrated that diagnostic assays to detect *C. burnetii* based on *IS1111* alone can lead to the misidentification of CLEs. To confirm identity and enable phylogenetic analysis, a subset of samples that tested positive in the *IS1111* assay was further characterized by nested PCR targeting the 16S rRNA, *groEL*, and *rpoB* genes.

The main limitation of this study is that ticks were collected from only three regions within Hebei Province, making it impossible to fully characterize the overall prevalence of CLE among ticks in China. Furthermore, CLE was only detected in *Ha. longicornis* ticks in Hebei; no other tick species were collected. Therefore, further research is needed to determine whether other tick species in Hebei carry CLE, which will require expanding the collection area and increasing tick species diversity.

## 5. Conclusions

In this study, CLE in *Ha. longicornis* from Hebei Province, China, was identified, reinforcing their widespread association with ticks globally. Phylogenetic analysis revealed two distinct CLE clades. Whether CLE poses a public or veterinary health risk remains unknown; given the lack of evidence for pathogenicity or zoonotic potential, further research—particularly on their ability to infect humans and livestock—is essential to determine whether these bacteria pose a threat to public health.

## Figures and Tables

**Table 1:** Primer sequences used in this study.

Target Gene	Primer	Oligonucleotide Sequences (5′–3′)	Reference
*IS1111*	QBT1	TATGTATCCACCGTAGCCAGTC	[17]
	QBT2	CCCAACAACACCTCCTTATTC	
	QBTN3	AAGCGTGTGGAGGAGCGAACC	[18]
	QBTN4	CTCGTAATCACCAATCGCTTCGTC	
16S rRNA	16S-F1	CGTAGGAATCTACCTTRTAGWGG	[19]
	16S-F	TGAGAACTAGCTGTTGGRRAGT	
	16S-R	GCCTACCCGCTTCTGGTACAATT	
*groEL*	CoxGrF1	TTTGAAAAYATGGGCGCKCAAATGGT	[20]
	CoxGrR2	CGRTCRCCAAARCCAGGTGC	
	CoxGrF2	GAAGTGGCTTCGCRTACWTCAGACG	
	CoxGrFR1	CCAAARCCAGGTGCTTTYAC	
*rpoB*	CoxrpoBF2	GGGCGNCAYGGWAAYAAAGGSGT	
	CoxrpoBR1	CACCRAAHCGTTGACCRCCAAATTG	
	CoxrpoBF3	TCGAAGAYATGCCYTATTTAGAAG	
	CoxrpoBR3	AGCTTTMCCACCSARGGGTTGCTG	

**Table 2:** Positive results for *Coxiella*-like endosymbiont.

Gene	Collecting Area		
	Chengde	Baoding	Cangzhou
*IS1111*	59/623 (9.47%)	5/204 (2.45%)	14/120 (11.67%)
16S rRNA	48/59 (81.36%)	2/5 (40%)	6/14 (42.86%)
*groEL*	45/59 (76.27%)	3/5 (60%)	5/14 (35.71%)
*rpoB*	52/59 (88.14%)	2/5 (40%)	4/14 (28.57%)

## Data Availability

Databases supporting the conclusions of this article are included in the article. All the *IS1111* gene sequences obtained in this study have been submitted to GenBank under the accession numbers PZ276418-PZ276495. All the 16S rRNA gene sequences obtained in this study have been submitted to GenBank under the accession numbers PZ269377-PZ269432. All the *rpoB* gene sequences obtained in this study have been submitted to GenBank under the accession numbers PZ276549-PZ276606. All the *groEL* gene sequences obtained in this study have been submitted to GenBank under the accession numbers PZ276496-PZ276548.
